# Treatment of Fracture of the Lower Jaw by Interdental Splints

**Published:** 1868-09

**Authors:** Thomas Brian Gunning


### ARTICLE II.

### *Treatment of Fracture of the Lower Jaw by Interdental Splints.*

By Thomas Brian Gunning.

(Concluded.)

Case 8—I applied the wings of Fig. 3 in the case of a distinguished statesman in Washington, whose jaw was fractured on both sides between the bicuspids.

The injury was caused by falling from a carriage, April 5th, 1865. Unsuccessful attempts had been made to hold the jaw in place by bandages, and also with ligatures on the teeth, by the Surgeons first called to the case. On the 14th, the patient, while asleep, was attacked by an assassin, and a cut inflicted which reached from under the right zygoma to the left of the trachea. Steno's duct was severed, and the right fracture laid open externally, the bone being also much exposed in the mouth from the original injury.

In accordance with the letter of April 14th, from Dr. Wm. Whelan, chief of the Naval Bureau of Medicine and Surgery, in answer to one by surgeon Bache, Director of the Naval Labratory, suggesting the use of an interdental splint, and telegrams of the 15th, urging me to come on at once, I started for Washington, and reached the patient's house at twelve, noon, on April 16th. Attending Surgeon, Basil Norris, U. S. A., informed me that the jaw was fractured on the right side, between the bicuspid teeth, and also in the ramus of the same side; that the jaw had been bandaged against the upper gum, but this proving insupportable to the patient the bandages were removed. Upon examination I found discoloration caused by the accident still remaining on the right side of the face. A cut (inflicted in the attempted assassination) commenced under the zygoma, passed forward about three inches, then downward and backward an equal distance, to the lower border of the jaw, from whence it crossed over the front of the throat to the left of the trachea. On the skin its first direction fell somewhat from a horizontal line, the second passed down at a little less than a right angle to the first, while the third went forward and downward. These three divisions, of nearly equal length, appeared to have been made by one sweep of the knife. Across the throat the wound was superficial, but above the border of the jaw it grew deeper, as it *split* the cheek—the point of the knife making no entrance into the mouth, except so far as it may be considered to have done so by laying open the right fracture externally, the gum being already lacerated internally from the great displacement of the bone following upon the original injury. The knife was evidently aimed at the throat, but the head being thrown over (the arm being useless) the cheek and jaw received the brunt of the blow. No arteries have been ligatured. The wound was neatly sewed up, and healing by first intention, except immediately under the fracture. The swelling and stiffness made the examination difficult, but the ramus proved to be uninjured. There was, however, a second fracture, but on the other side of the mouth, the jaw being fractured on both sides between the bicuspids. The jaw contained all the ten forward teeth. The right wisdom tooth and root of the left were all that remained back of the bicuspids. The part in front, containing eight teeth, was drawn down out of place, while the right back fragment, with the wisdom tooth and second bicuspid, was drawn up, showing its fractured end white and bare. The fracture was square across, vertical and smooth, and the parts were separated vertically over a quarter of an inch when at rest, sometimes much more. On the left side, the first bicuspid fell forward and downward from the second, one-quarter of an inch. This fracture passed forward somewhat in descending. Here the bone could not be seen, as the gum had separated from both teeth and lay swollen over it. Pus discharged profusely from both fractures. The gum was pale and flaccid, in keeping with the general condition of the patient. The upper jaw was entirely without teeth. Deeming it important to set the exposed bone in place as early as possible, and also to give the patient time to recuperate—as he had already been subjected, during the morning, not only to a relation of the President's death, but to much that was said and written upon the subject—I obtained the patient's artificial teeth, intending to cut out the front teeth, and tie the lower natural canines to the upper artificial ones. In this way the back fragments would have been kept down in place, and in return would have held the artificial teeth up against the roof of the mouth. They could have been used therefore to support the front of the lower jaw temporarily, without assistance from bandages, which were not only inadmissible in consequence of the wounds, etc., but would have increased the tendency to necrosis by interfering with the circulation. But the patient's experience with the teeth had not been such as encouraged him that he could bear them in his mouth. It was therefore necessary to leave the parts as they were until the next morning.

In the afternoon, while explaining the treatment proper for the case to Dr. Whelan, I also stated my unwillingness to commence, except with the understanding that I should control it entirely.

April 17th. Was informed by Surgeon Norris, that the friends of the patient were unwilling to have the splint fitted to the jaw at present, and that the surgeons agreed with them.

Upon giving my views to the contrary, Dr. Norris came over to my opinion. I consented to wait until the following morning, when it was finally decided not to proceed in the matter. I protested, in vain, but promised to return when sent for.

April 28th. Arrived in Washington; Surgeon-General Barnes informed me that the jaw was more displaced, but the patient otherwise much improved. I found the sensation of the right side of the forehead, face and lips deficient. The separation of the inferior dental nerve by the displacement of the bone, and of branches of the facial nerve, by the knife, did not seem sufficient to account for it. There was also irregular motion in the right eye. The front of the jaw was lower, and the right back fragment showed its alveolus to a greater extent. There were no indications of any tendency to the union on either side. The fragment could be put precisely in place, no splinters or anything else intervening. There was little swelling, but great discharge of pus. Took wax impression of the upper jaw, and removed the tartar from the lower teeth.

April 29th. I set the jaw, and held it in place by wire and silk ligatures, as described on page 54, June number. Took wax impression of the teeth and gum, and obtained the bite directly from the teeth, etc.*

April 30th. Patient felt much relieved, as the ligatures held the front of the jaw up well. Tried in a gutta-percha splint, arranged the wings in it, removed it carefully from the mouth, placed the upper and lower casts and female screws in it, and set them in a vulcanizing flask.

Although the front of the jaw containing the eight forward teeth was greatly displaced (before the setting), the silk and wire ligatures held well until May 2d, when they were removed and the splint applied. It was of hard vulcanized rubber, covered the roof of the mouth and adjacent gum, inclosed all the lower teeth, and went down over the gum on the outside somewhat. The opening in front was seven-eighths of an inch wide, and half an inch high in the centre, the wings preventing any more room sideways, as they were set clear of the commissure of the lips. To have given more room in the height, by depressing the lower jaw would have made it very difficult to prevent the saliva from overflowing at the lips. Upon putting in the splint the breathing was

---

* In doing this, and in making the splint, I was assisted by Mr. J. Adams Bishop, who accompanied me from New York.

very spasmodic for several minutes, but this soon passed off, and I screwed it fast to the lower teeth. They held it against the upper gum for the first night, but after that a cap, with adjuncts, as in Fig. 3, was worn to support the splint. The upper wings only were used, as the lower jaw was held up in the splint by screws passing into the lower canines. The mental band was consequently not applied, although the lower wings were left on in case of need. The upper wings being kept clear of the zygomas, the parts around the jaw and face were left free from pressure—this being important in order that the vascular and nervous circulation should be unimpeded. After giving the excellent army nurses who were in attendance upon the patient full direction for keeping the splint clean in the mouth, and properly balanced by the cap, which I had fitted to the head, I left Washington, May 3d.

Arrived in Washington again on the 8th, having received a telegram saying that the patient was suffering much pain. Found him quite comfortable, talking freely and much encouraged. Saliva had accumulated several times in the cheek, but had been let out by lancing externally. The splint had been kept quite clean, and as every thing was going on well I left on the 9t h

June 11th. Saw the patient again. The left side appeared to be well united, but the right gave no indication of union, although the wound under it was nearly closed, the last of several pieces of bone having been removed some days before. I promised to remove the splint in four weeks from that date to examine the parts.

This splint held the jaw firm for sixty-eight days, when I removed it.

There was good union on the left side, but the right fracture was still ununited. For this, however, I was prepared, as the bone had been exposed so much during the twenty-four days which elapsed before I set it, and the saliva from the right parotid gland had discharged through the fracture from a short time after the attack. These unfavorable conditions, with other depressing circumstances, associated with an enfeebled condition from loss of blood, had been followed by necrosis of the ends of the bone on that side, and several pieces had come away externally during the first six weeks from the time the splint was applied, and also a long piece from the inside of the jaw on the left side.

I now removed the necrosed alveolus of the second bicuspid, but left the tooth in, as it appeared to have healthy connection with the lower part of its socket. The other teeth had grown firm. The splint had not been off the jaw a moment since its first application, and therefore little examination had been made internally, but external appearances had indicated that the saliva followed the course taken by the point of the knife. At this time, July 9th, Steno's duct proved to be completely closed. I could not pass the smallest probe even into its mouth, and the saliva discharged wholly through the ununited fracture.

Upon removing the first splint I immediately put another upon the teeth. This splint was ready for application, having been made on a cast taken from the original impression. This second splint was like Fig. 1. It covered all the teeth and gum, and was worn from July 9th to August 4th, when I removed it and put on a splint which allowed all the teeth to be seen, except the wisdom tooth on the right and the root on the left side, upon which the splint rested. This splint was worn screwed to the canines, until the beginning of September‘ four months from the application of the first splint. I saw the patient several times during the month of October. The jaw seemed to be getting firmer on the right side. On the left it was then quite strong and all precisely in place.

The patient talked freely while wearing the splints, except for a few days at the commencement. From the time the second was applied the jaw has been used for eating.

In letter to me of March 29, 1866, the patient says: "The whole jaw moves quite well and firmly. Thus at last I begin to regard my cure in that respect complete."

I have not seen it myself since October, 1865, therefore cannot speak of it by personal observation.

Of the splints spoken of in this paper, with their wings and other appliances, I am enabled to give most decided assurances of their perfect adaptability to the purpose for which they were devised.

Having personally experienced their great advantage, and believing them to be superior to all other treatment, I have endeavored to make the application of them as easy as possible, desiring that others, whether practioners or patients, may have the benefit of their use, when necessary.

---

